# RIOX1-demethylated cGAS regulates ionizing radiation-elicited DNA repair

**DOI:** 10.1038/s41413-022-00194-0

**Published:** 2022-02-24

**Authors:** Yanxuan Xiao, Jingyi Li, Xiaoyu Liao, Yumin He, Tao He, Cuiping Yang, Lu Jiang, So Mi Jeon, Jong-Ho Lee, Yongbin Chen, Rui Liu, Qianming Chen

**Affiliations:** 1 State Key Laboratory of Oral Diseases, National Clinical Research Center for Oral Diseases, Chinese Academy of Medical Sciences Research Unit of Oral Carcinogenesis and Management, West China Hospital of Stomatology, Sichuan University, Chengdu, Sichuan 610041 China; 2 The Second Affiliated Hospital of Chengdu Medical College, China National Nuclear Corporation 416 Hospital, Chengdu, Sichuan 610051 China; 3 School of Biological Sciences and Technology, Chengdu Medical College, Chengdu, 610599 China; 4 Department of Cardio-thoracic Surgery, the Second Affiliated Hospital of Chengdu Medical College, China National Nuclear Corporation 416 Hospital, Chengdu, Sichuan 610051 China; 5 Key Laboratory of Animal Models and Human Disease Mechanisms of Chinese Academy of Sciences Yunnan Province, Kunming Institute of Zoology, Kunming, Yunnan 650223 China; 6 Department of Health Sciences, The Graduate School of Dong-A University, Busan, 49315 Republic of Korea; 7 Department of Biological Sciences, Dong-A University, Busan, 49315 Republic of Korea; 8 Center for Excellence in Animal Evolution and Genetics, Chinese Academy of Sciences, Kunming, Yunnan 650223 China

**Keywords:** Bone, Metabolic diseases

## Abstract

Exposure to radiation causes DNA damage; hence, continuous surveillance and timely DNA repair are important for genome stability. Epigenetic modifications alter the chromatin architecture, thereby affecting the efficiency of DNA repair. However, how epigenetic modifiers coordinate with the DNA repair machinery to modulate cellular radiosensitivity is relatively unknown. Here, we report that loss of the demethylase ribosomal oxygenase 1 (RIOX1) restores cell proliferation and reduces cell death after exposure to ionizing radiation. Furthermore, RIOX1 depletion enhances homologous recombination (HR) repair but not nonhomologous end-joining (NHEJ) repair in irradiated bone marrow cells and oral mucosal epithelial cells. Mechanistic study demonstrates that RIOX1 removes monomethylation at K491 of cyclic GMP-AMP synthase (cGAS) to release cGAS from its interaction with the methyl-lysine reader protein SAGA complex-associated factor 29 (SGF29), which subsequently enables cGAS to interact with poly(ADP-ribosyl)ated poly(ADP-ribose) polymerase 1 (PARP1) at DNA break sites, thereby blocking PARP1-mediated recruitment of Timeless. High expression of RIOX1 maintains cGAS K491me at a low level, which impedes HR repair and reduces cellular tolerance to ionizing radiation. This study highlights a novel RIOX1-dependent mechanism involved in the non-immune function of cGAS that is essential for the regulation of ionizing radiation-elicited HR repair.

## Introduction

Exposure to ionizing radiation may cause both acute symptoms and long-term health effects.^1^ Among mammalian organs, the bone marrow is the most vulnerable to ionizing radiation-induced damage due to the rapid turnover of immature hematopoietic cells. While suppression of mature blood cells is a common consequence in patients who receive radiotherapy, mortality is believed to be caused by the exhaustion of both hematopoietic progenitor cells and primitive hematopoietic stem cells in the bone marrow.^2^

Ionizing radiation results in various lesions in both genomic and mitochondrial DNA, among which double-strand breaks (DSBs) are arguably the most harmful.^3^ DSBs are repaired via two main pathways, homologous recombination (HR) and nonhomologous end-joining (NHEJ).^4^ HR repair of damaged sites involves template-guided DNA extension, which guarantees error-free repair by restoring sequence information from the templates. In contrast, in NHEJ repair, the two broken ends of chromosomes are directly ligated in an error-prone manner.^5^

Jumonji C domain (JMJD)-containing demethylase family, which includes over 30 members, plays an important role in regulating DNA repair.^6–8^ Ribosomal oxygenase 1 (RIOX1) removes mono- or trimethylation from histone 3 lysine 4 (H3K4me1 or H3K4me3, respectively), while it has weaker activity toward methylated H3K36.^9^ However, whether RIOX1 is involved in DNA repair upon ionizing radiation-induced damage is largely undefined. In the current study, we report a novel RIOX1-mediated mechanism that modulates the efficiency of HR repair. We show that depletion of RIOX1 in mouse bone marrow cells restores cell viability and decreases cell death upon exposure to ionizing radiation. Loss of RIOX1 enhances HR repair but not NHEJ repair in irradiated cells. Furthermore, we demonstrate that RIOX1 abolishes the monomethylation of K491 in cyclic GMP-AMP synthase (cGAS) (corresponding to K506 in human cGAS), which disrupts the interaction between cGAS and the methyllysine reader protein SAGA complex-associated factor 29 (SGF29). cGAS without K491me binds to poly(ADP-ribosyl)ated poly(ADP-ribose) polymerase 1 and abrogates the recruitment of Timeless to DNA break sites, thereby blocking HR repair. RIOX1 is highly expressed in bone marrow cells with a relatively low level of cGAS K491me, consistent with the high radiosensitivity of bone marrow cells.

## Results

### RIOX1 decreases cell viability and inhibits HR repair in bone marrow cells after ionizing radiation

**Fig. 1:**
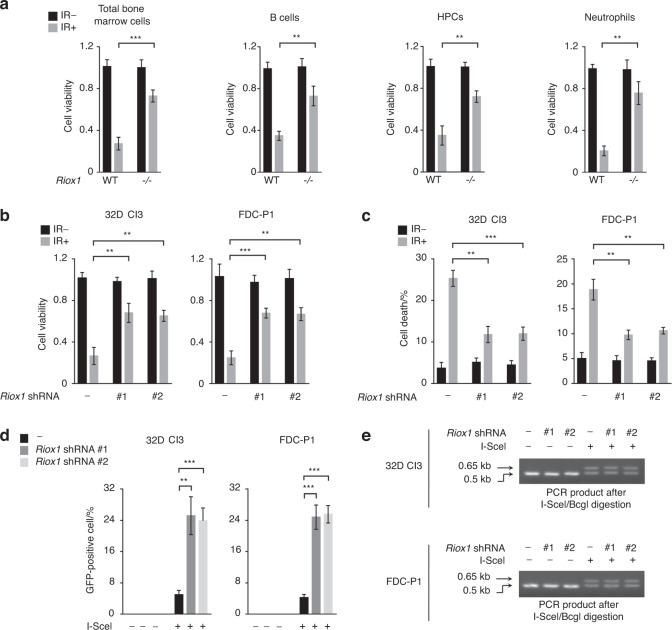
RIOX1 decreases cell viability and inhibits HR repair in bone marrow cells after ionizing radiation. The data are shown as the mean ± SD of three independent assays. **P < 0.01; ***P < 0.001. **a** WT or *Riox1*^-/-^ mice were treated with 9 Gy ionizing radiation. The indicated bone marrow cells were isolated 16 h after irradiation, and cell viability was measured. **b**, **c** 32D Cl3 and FDC-P1 cells expressing *Riox1* shRNAs were treated with 10 Gy ionizing radiation. Cell viability (**b**) and cell death (**c**) were evaluated 48 h after irradiation. Cell viability data were normalized to the untreated group. 32D Cl3 and FDC-P1 cells expressing *Riox1* shRNAs were transfected with an I-SceI expression vector. HR repair was tested 72 h after transfection (**d**). NHEJ repair was examined 1 h after irradiation (**e**)

To evaluate the effects of RIOX1 on bone marrow cells after ionizing radiation, RIOX1 knockout mice were examined. Adult RIOX1 knockout mice were viable and fertile. We noted an increased body weight and length at birth and at 8 weeks of age (Fig. S1A and S1B). Loss of RIOX1 resulted in prolonged survival (Fig. S1C) and attenuated symptoms of diarrhea (Fig. S1D) after irradiation. We further obtained bone marrow cells from the tibiae of the irradiated mice and ultimately isolated B cells, hematopoietic progenitor cells (HPCs) and neutrophils. As expected, ionizing radiation largely decreased the viability of total bone marrow cells and all three cell subsets tested (Fig. 1a). Notably, the irradiation-induced decrease in cell viability was substantially reversed in bone marrow cells isolated from RIOX1 knockout mice compared to those isolated from their wild-type (WT) counterparts (Fig. 1a). Furthermore, we treated the mouse bone marrow myeloid 32D Cl3 cell line, which expresses CD11a and CD11b^10^ but not the fibroblast marker vimentin or epithelial cell marker E-Cad (Fig. S1E), with 10 Gy ionizing radiation. Consistent with the above findings, knockdown of endogenous RIOX1 in 32D Cl3 cells by two distinct shRNAs substantially attenuated the irradiation-induced cell viability suppression and cell death (Fig. 1b and c). Similar results were obtained in the CD34- and Thy-1-expressing mouse bone marrow myeloid cell line FDC-P1^11^ (Figs. 1b, c and S1E).

Effective DNA repair is important to fix DNA damage.^12^ Strikingly, knockout of RIOX1 largely eliminated the irradiation-induced DNA fragments in bone marrow cells, as revealed by the shortened tail-like smear in the comet assay (Fig. S1F), suggesting that RIOX1 is likely involved in modulating irradiation-elicited DNA damage. To determine the impact of RIOX1 on HR and NHEJ repair, the endonuclease I-SceI was expressed to generate a DSB site in an exogenously introduced DR-GFP locus. DNA repair through HR removed the I-SceI cut site and generated a BcgI cut site, leading to the expression of DR-GFP; in contrast, NHEJ repair resulted in a 0.65-kb PCR product that was resistant to both I-SceI and BcgI digestion (Fig. S1G).^13^ We found that expression of I-SceI induced GFP expression in approximately 5% of 32D Cl3 and FDC-P1 cells, and this GFP expression was appreciably enhanced upon RIOX1 knockdown (Figs. 1d and S1H). The different amounts of GFP-expressing cells were presumed to reflect the different HR repair efficiencies, since a similar I-SceI cleavage efficiency was detected in *Riox1* shRNA-treated and untreated cells before DNA repair was initiated (Fig. S1I). However, loss of RIOX1 minimally affected NHEJ repair, as revealed by the comparable amounts of the 0.65-kb PCR product (Fig. 1e). These results suggest that RIOX1 decreases cell viability and inhibits HR repair in bone marrow cells after ionizing radiation.

### RIOX1 binds to cGAS and demethylates cGAS K491me

To explore the mechanisms underlying the RIOX1-mediated repression of HR repair, we depleted RIOX1 activity in 32D Cl3 cells by replacing endogenous WT RIOX1 with the shRNA-resistant Flag-RIOX1 H302 A/H367A mutant (corresponding to human RIOX1 H339A/H404A), which lacks lysine demethylase activity (Fig. S2A).^9^ Loss of RIOX1 activity induced an increase in HR repair efficiency similar to that caused by RIOX1 knockdown (Fig. S2A), suggesting that demethylase activity is required for RIOX1-mediated repression of HR repair. Surprisingly, reconstitution of RIOX1 expression with this mutant only evoked limited accumulation of H3K4me, H3K4me3 and H3K36me3 (Fig. S2B), which might be interpreted in the context of previous reports showing that methylation of these sites can also be removed by a couple of other lysine demethylases.^14^ Furthermore, human histone H3 is encoded by several genes and expressed as multiple variants, which are highly conserved with only a few nonconserved residues.^15^ In line with previous reports,^16,17^ targeting H3.3B mRNA (gene symbol: H3f3b) reduced total H3 expression by approximately 80% in 32D Cl3 cells, as revealed by analysis with an antibody that recognizes all H3 variants, suggesting that H3.3B is the major histone H3 isoform (Fig. S2C). Strikingly, reconstitution of H3.3B expression with the shRNA-resistant H3.3B K4R or K36R mutant failed to abolish the increased HR repair in RIOX1-depleted 32D Cl3 cells (Fig. S2C). Together, these results suggest that a nonhistone substrate is likely involved in RIOX1-mediated regulation of HR repair.

**Fig. 2:**
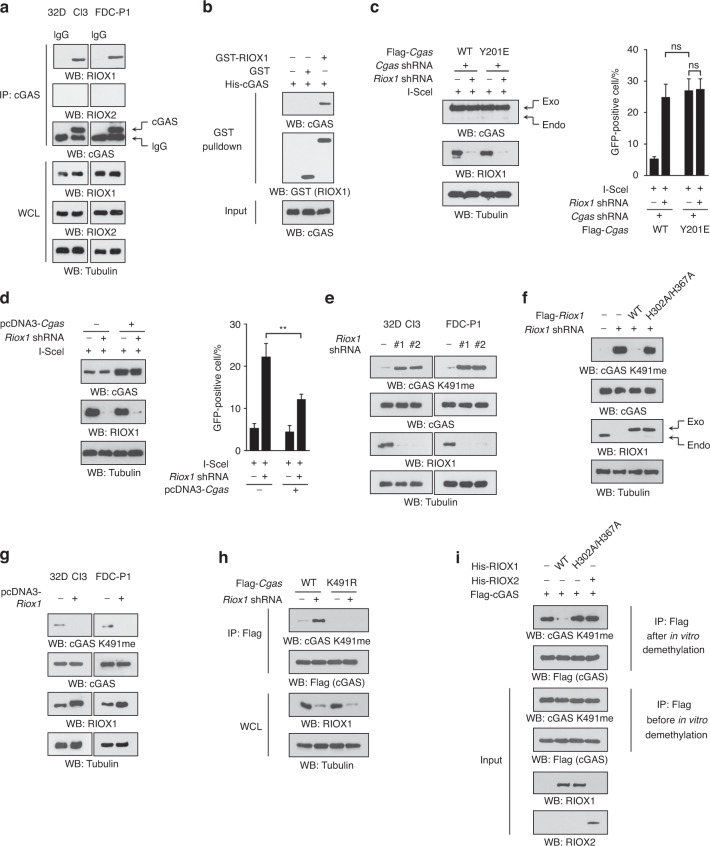
RIOX1 binds to and demethylates cGAS K491me. **a–i** Immunoblot analysis was performed. **a** Coimmunoprecipitation was performed using 32D Cl3 and FDC-P1 cell samples. **b** Bacterially purified GST-RIOX1 or GST protein was mixed with purified His-cGAS protein. A GST pulldown assay was performed. **c** 32D Cl3 cells expressing *Riox1* shRNA, *Cgas* shRNA, WT Flag-*Cgas*, or Flag-*Cgas* Y201E were transfected with an I-SceI expression vector. HR pathway activity was measured. The *Cgas* shRNA targeted the noncoding region. The data are shown as the mean ± SD of three independent assays. ns, not significant. **d** 32D Cl3 cells expressing *Riox1* shRNA or *Cgas* were transfected with an I-SceI expression vector. HR pathway activity was measured. The data are shown as the mean ± SD of three independent assays. **P < 0.01. **e** 32D Cl3 and FDC-P1 cells were transduced with *Riox1* shRNAs. **f** 32D Cl3 cells were transduced with *Riox1* shRNA, WT Flag-*Riox1* or Flag-*Riox1* H302A/H367A. The *Riox1* shRNA targeted the noncoding region. **g** 32D Cl3 and FDC-P1 cells were transduced with a *Riox1* overexpression vector. **h** 32D Cl3 cells were transfected with *Riox1* shRNA, WT Flag-*Cgas*, or Flag-*Cgas* K491R. Immunoprecipitation was performed. **i** Flag-cGAS protein was purified from *Riox1*-depleted 32D Cl3 cells and incubated with bacterially purified WT His-RIOX1, His-RIOX1 H302A/H367A, or WT His-RIOX2 protein for an in vitro demethylation assay

Next, we pulled down Flag-tagged RIOX1 protein from 32D Cl3 cells, and cGAS was detected in the RIOX1 immunoprecipitates by mass spectrometry (Fig. S3A). cGAS was previously found to translocate into the nucleus upon ionizing radiation and block HR repair.^18^ The interaction between endogenous RIOX1 and cGAS was confirmed by coimmunoprecipitation in both 32D Cl3 and FDC-P1 cells (Fig. 2a). An in vitro protein binding assay showed that bacterially purified cGAS protein could be pulled down by purified GST-RIOX1 protein, suggesting that these two proteins directly interact with each other (Fig. 2b). However, no interaction between cGAS and RIOX2, which shares 40% homology with RIOX1, was detected (Fig. 2a). Furthermore, reconstitution of cGAS expression with the cGAS Y201E mutant (corresponding to human cGAS Y215E), which is resistant to ionizing radiation-induced translocation into the nucleus,^18^ enhanced HR repair in 32D Cl3 cells, and HR repair could not be further increased by RIOX1 knockdown (Fig. 2c). In contrast, overexpression of cGAS substantially reduced RIOX1 depletion-induced HR repair (Fig. 2d).

To determine whether RIOX1 affects the methylation status of cGAS, we analyzed cGAS protein extracted from RIOX1-depleted 32D Cl3 cells and identified monomethylation of cGAS K491, which is evolutionarily conserved among species (corresponding to human cGAS K506, Fig. S3B). Additionally, the residues adjacent to K491 are analogous to those adjacent to H3K4 or and H3K36 (Fig. S3B). To quantify this methylation, we produced an antibody that specifically recognizes the mouse cGAS K491me peptide but not the unmodified K491, K491me2 or K491me3 peptide (Fig. S3C). By using this antibody, we found that cGAS K491me highly accumulated in cells with depletion of RIOX1 expression or activity (Fig. 2e and f), although the expression level of endogenous cGAS was unchanged (Fig. 2e and f). In contrast, knockdown of JMJD1A, JMJD1B, or LSD1, all of which are capable of removing H3K4me or H3K36me, did not affect the level of cGAS K491me in 32D Cl3 cells (Fig. S3D), suggesting that these enzymes are not responsible for cGAS K491me demethylation. Immunoblotting showed that this modification was abolished by incubation with an excess of the cGAS K491me peptide, suggesting the good specificity of the anti-cGAS K491me antibody (Fig. S3E). Consistent with this finding, overexpression of RIOX1 or the cGAS K491R mutant abrogated this modification (Figs. 2g and h). However, ionizing radiation had minor effects on the level of cGAS K491me in both RIOX1-expressing and RIOX1-depleted cells (Fig. S3F).

To verify whether RIOX1 directly catalyzes the demethylation of cGAS K491me, Flag-cGAS protein was purified from RIOX1-depleted 32D Cl3 cells and was then subjected to an in vitro demethylation assay. The K491me modification was clearly detected in the purified cGAS protein (Fig. 2i). Moreover, incubation with bacterially purified WT RIOX1 protein but not the purified RIOX1 H302A/H367A mutant or the RIOX2 protein resulted in erasure of cGAS K491me (Fig. 2i). These results suggest that RIOX1 binds to and demethylates cGAS K491me.

### RIOX1 overrides SET7-mediated methylation and governs cGAS K491me

**Fig. 3:**
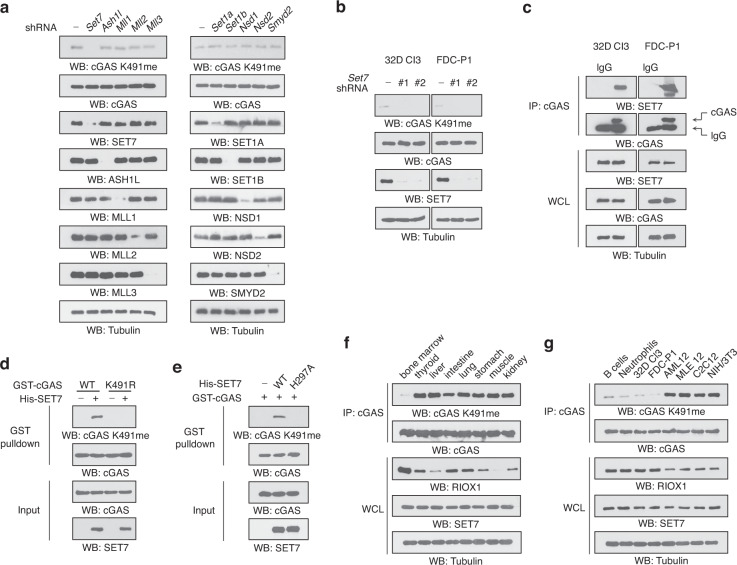
RIOX1 overrides SET7-mediated methylation and governs cGAS K491me. **a–g** Immunoblot analysis was performed. **a** 32D Cl3 cells were transduced with shRNA targeting *Set7, Ash1I, MII1, MII2, MII3, Set1a, Set1b, Nsd1, Nsd2*, or *Smyd2*. **b** 32D Cl3 and FDC-P1 cells were transduced with *Set7* shRNAs. **c** Coimmunoprecipitation was performed using 32D Cl3 and FDC-P1 cell samples. **d** Purified WT GST-cGAS or GST-cGAS K491R protein was incubated with purified His-SET7 protein for an in vitro methylation assay. A GST pulldown assay was performed. **e** Purified GST-cGAS protein was incubated with purified WT His-SET7 or His-SET7 H297A protein for an in vitro methylation assay. A GST pulldown assay was performed. **f**, **g** Immunoprecipitation was performed using lysates of the indicated mouse tissues or cells

We sought to identify the potential methyltransferases responsible for cGAS K491me by screening a set of reported enzymes. Knockdown of only SET domain-containing lysine methyltransferase 7 (SET7) reduced the cellular level of cGAS K491me (Fig. 3a). This observation was confirmed by treating 32D Cl3 and FDC-P1 cells with two distinct *Set7* shRNAs (Fig. 3b). SET7 protein was found in the cGAS immunoprecipitates, indicating the interaction between endogenous SET7 and cGAS (Fig. 3c). Furthermore, an in vitro protein methylation assay revealed that incubation with bacterially purified His-SET7 protein induced K491me of GST-cGAS, while this methylation was blocked when the methylatable lysine-substituted cGAS K491R mutant or the catalytically dead SET7 H297A mutant was used in the reaction system (Fig. 3d, e). These results suggest that SET7 methylates cGAS at K491.

To determine how RIOX1 and SET7 mutually regulate cGAS K491me in cells, we compared the level of cGAS K491me and the expression of RIOX1 and SET7 among various mouse organs. We found that, compared to other organs, the bone marrow showed an increased expression level of RIOX1 and a reduced level of cGAS K491me (Fig. 3f). However, SET7 was ubiquitously expressed in different organs, with minor changes (Fig. 3f). Consistent with this finding, RIOX1 expression was enriched in 32D Cl3 cells, FDC-P1 cells and mouse bone marrow-derived B cells and neutrophils with a decreased level of cGAS K491me; in contrast, lower RIOX1 expression level and a correspondingly increased cGAS K491me level were found in the normal mouse liver cell line AML12, lung epithelial cell line MLE 12, myoblast line C2C12 and fibroblast line NIH/3T3 (Fig. 3g). These results suggest that RIOX1 expression governs the level of cGAS K491me.

### RIOX1-mediated demethylation of cGAS K491me facilitates cGAS binding to PARP1

cGAS recognizes double-stranded DNA, including endogenous genomic DNA fragments, to activate innate immune responses.^19^ However, loss of RIOX1 has limited impact on ionizing radiation-induced phosphorylation of TANK-binding kinase 1 (TBK1) at S172 (Fig. S4A), a hallmark of cGAS activation^20^. This finding was further supported by the observation that herring testis (HT)-DNA equally elicited the catalytic activity of purified cGAS protein with or without SET7-mediated K491me (Fig. S4B). Furthermore, the cellular fractionation assay showed a similar subcellular distribution of cGAS in untreated and *Riox1* shRNA-treated cells (Fig. S4C), suggesting that RIOX1 does not regulate the cytoplasmic-nuclear shuttling of cGAS.

**Fig. 4:**
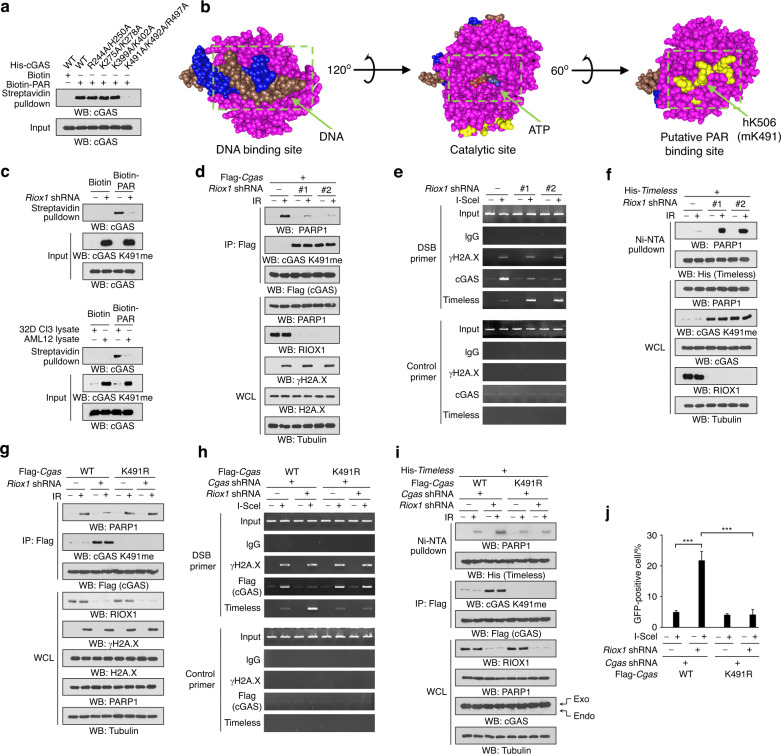
RIOX1-mediated demethylation of cGAS K491me facilitates cGAS binding to PAR. **a**–**d**, **f**, **g**, **i** Immunoblot analysis was performed. **a** Equal amounts of biotin-PAR were incubated with bacterially purified WT His-cGAS or the indicated mutant protein, and a streptavidin pulldown assay was performed. **b** The DNA binding site, catalytic domain and putative PAR binding motif in the human cGAS structure (PDB ID: 6CTA) are shown. The basic and hydrophobic residues in the putative PAR binding motif are shown in yellow. **c** Equal amounts of biotin-PAR were incubated with lysates of untreated or *Riox1*-depleted 32D Cl3 cells (upper panel). Equal amounts of biotin-PAR were incubated with lysates of AML12 or 32D Cl3 cells (bottom panel). The cell lysate input was used for normalization of the cGAS expression level. A streptavidin pulldown assay was performed. **d** 32D Cl3 cells expressing *Riox1* shRNA or Flag-*Cgas* were treated with 10 Gy ionizing radiation. Immunoprecipitation was performed. The immunoprecipitates were treated with an excess of purified PARG protein before being subjected to immunoblot analysis. **e** 32D Cl3 cells expressing *Riox1* shRNA were transfected with an I-SceI expression vector. ChIP-PCR was performed. **f** 32D Cl3 cells expressing *Riox1* shRNA or His-*Timeless* were treated with 10 Gy ionizing radiation. A Ni-NTA pulldown assay was performed. The precipitates were treated with an excess of purified PARG protein before being subjected to immunoblot analysis. **g** 32D Cl3 cells expressing *Riox1* shRNA, WT Flag-*Cgas* or Flag-*Cgas* K491R were treated with 10 Gy ionizing radiation. Immunoprecipitation was performed. The immunoprecipitates were treated with an excess of purified PARG protein before being subjected to immunoblot analysis. 32D Cl3 cells expressing *Riox1* shRNA, WT Flag-*Cgas* or Flag-*Cgas* K491R were transfected with an I-SceI expression vector. ChIP-PCR was performed 30 h after transfection **h**. HR pathway activity was measured 72 h after transfection **j**. The data are shown as the mean ± SD of three independent assays. ***P < 0.001. **i** 32D Cl3 cells expressing *Riox1* shRNA, WT Flag-*Cgas*, Flag-*Cgas* K491R, or His-*Timeless* were treated with 10 Gy ionizing radiation. A Ni-NTA pulldown assay and immunoprecipitation were performed. The immunoprecipitates were treated with an excess of purified PARG protein before being subjected to immunoblot analysis

Ionizing radiation-induced nuclear cGAS interacts with PARP1 by binding with poly(ADP ribose) (PAR) chains and hinders PARP1-mediated recruitment of Timeless, thereby inhibiting HR repair. PAR can bind to proteins through a two-section motif that contains a basic amino acid-enriched N-terminus and a cluster of hydrophobic amino acids interspersed with basic residues at the C-terminus.^21^ Analyses of the cGAS protein sequence revealed four potential PAR binding sites: 238KFKRIPRGNPLSHFL252, 265KFRKIIKEEVKEIKDI280, 391KCCRKECLKLMKYLL405 and 483RKSKEFLSKKIEYER497. Replacement of the basic residues in the C-termini of these motifs with alanines revealed that only the mutations in the 483–497 aa region reduced the amount of cGAS pulled down by biotin-labeled PAR (Fig. 4a). Furthermore, the 483–497 aa region (corresponding to the 498–512 aa region in humans) did not overlap with the DNA binding region or the catalytic domain of cGAS (Fig. 4b), in line with the observation that the activity of K491-methylated cGAS was inducible by HT-DNA (Fig. S4B).

cGAS K491 is located within the 483–497 aa region, leading to the hypothesis that RIOX1-mediated cGAS K491me may affect cGAS binding with PAR. We performed an in vitro biotin-PAR pulldown assay using the lysates of untreated or RIOX1-depleted 32D Cl3 cells that contained equal amounts of cGAS protein. Indeed, equal amounts of biotin-PAR pulled down much less cGAS protein from the lysates of RIOX1-depleted 32D Cl3 cells than from those of untreated 32D Cl3 cells (Fig. 4c), suggesting that RIOX1 facilitates the interaction between cGAS and PAR. A similar result was obtained in the comparison between AML12 and 32D Cl3 cells, which have different endogenous RIOX1 expression and cGAS K491me levels (Figs. 3g and 4c). Furthermore, loss of RIOX1 reduced the amount of cGAS that was either associated with PARP1 in irradiated 32D Cl3 cells or recruited to I-Scel-induced DNA break sites (Figs. 4d, e and S4D). RIOX1 knockdown also promoted the irradiation-elicited translocation of Timeless to DNA break sites and the formation of the PARP1/Timeless complex (Fig. 4e, f). However, these *Riox1* shRNA-mediated effects were largely obliterated by reconstitution of cGAS expression with the cGAS K491R mutant (Fig. 4g−i). Accordingly, the cGAS K491R mutation also abolished the reinforcement of HR repair in RIOX1-depleted cells (Fig. 4j). These results suggest that RIOX1-mediated demethylation of cGAS K491me facilitates cGAS binding to PAR and impedes HR repair.

### SGF29 binds to K491me-bearing cGAS and prevents cGAS from binding to PAR

**Fig. 5:**
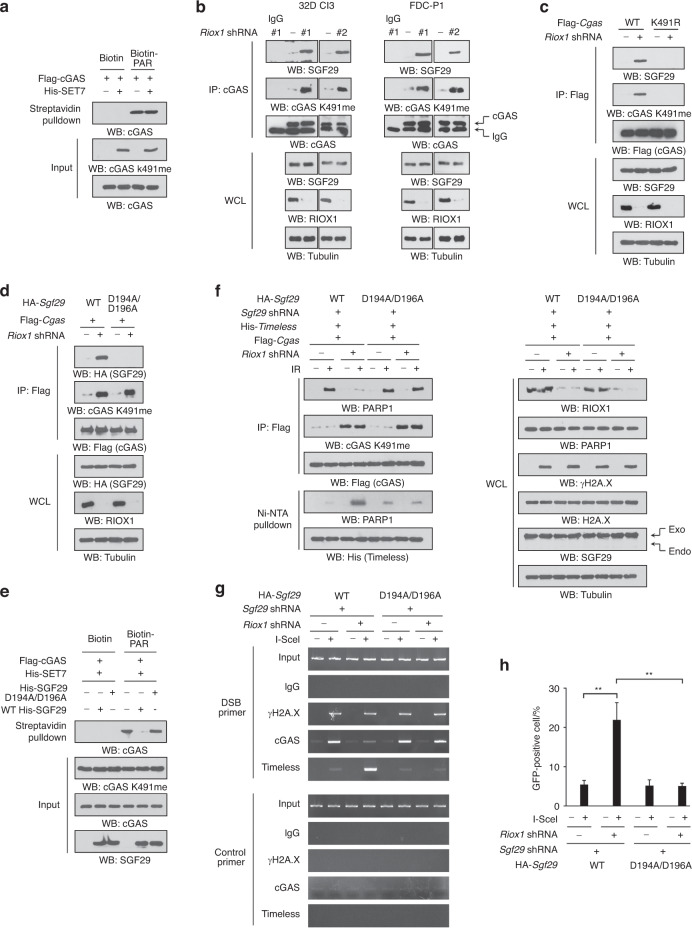
SGF29 binds to K491me-bearing cGAS and prevents cGAS from binding to PAR. **a–f** Immunoblot analysis was performed. **a** Purified Flag-cGAS protein was incubated with purified His-SET7 protein for an in vitro methylation assay and was then incubated with an equal amount of biotin-PAR. A streptavidin pulldown assay was performed. **b** 32D Cl3 and FDC-P1 cells were transduced with *Riox1* shRNAs. Coimmunoprecipitation was performed using 32D Cl3 and FDC-P1 cell lysates. **c** 32D Cl3 cells were transduced with *Riox1* shRNA, WT Flag-*Cgas*, or Flag-*Cgas* K491R. Immunoprecipitation was performed. **d** 32D Cl3 cells were transduced with *Riox1* shRNA, Flag-*Cgas*, WT HA-*Sgf29 or HA*-*Sgf29* D194A/D196A. Immunoprecipitation was performed. **e** Purified Flag-cGAS protein was incubated with purified His-SET7 protein for an in vitro methylation assay, incubated with purified WT or the indicated mutant SGF29 protein, and then incubated with an equal amount of biotin-PAR. A streptavidin pulldown assay was performed. **f** 32D Cl3 cells expressing *Riox1* shRNA, Flag-*Cgas*, His-*Timeless*, *Sgf29* shRNA, WT HA-*Sgf29*, or HA-*Sgf29* D194A/D196A were treated with 10 Gy ionizing radiation. A Ni-NTA pulldown assay and immunoprecipitation were performed. The immunoprecipitates were treated with an excess of purified PARG protein before being subjected to immunoblot analysis. The *Sgf29* shRNA targeted the noncoding region. **g**, **h** 32D Cl3 cells expressing *Riox1* shRNA, *Sgf29* shRNA, WT HA-*Sgf29*, or HA-*Sgf29* D194A/D196A were transfected with an I-SceI expression vector. ChIP-PCR was performed 30 h after transfection **g**. HR pathway activity was measured 72 h after transfection **h**. The data are shown as the mean ± SD of three independent assays. **P < 0.01

Lysine methylation frequently modulates protein functions via modulating the recruitment of binding partners.^22^ We found that SET7-mediated K491me on the bacterially purified cGAS protein failed to disrupt the interaction between cGAS and PAR, suggesting that other factors are likely involved in this process (Fig. 5a). SGF29, a methyllysine reader protein that can recognize methylated H3K4,^23^ was found when we revisited the aforementioned proteomic analyses to search for cGAS-associated proteins (Fig. S3A). Loss of RIOX1 increased cGAS K491me and accordingly promoted the interaction of endogenous SGF29 with cGAS in both 32D Cl3 and FDC-P1 cells (Fig. 5b). However, the interaction between these two proteins was abrogated by the cGAS K491R mutation (Fig. 5c) and the SGF29 D194A/D196A mutation, which conferred defective binding to methylated H3K4 (Fig. 5d).^24^

Since cGAS K491 is located within the PAR-binding motif, we reasoned that SGF29 may mask this motif when associated with cGAS, thereby preventing cGAS from binding to PAR. As expected, preincubation with purified His-SGF29 protein markedly abolished the biotin-PAR-mediated pulldown of purified K491me-bearing cGAS proteins (Fig. 5e). Consistent with this finding, reconstitution of SGF29 expression with the SGF29 D194A/D196A mutant restored the formation of the PARP1/cGAS complex, disrupted the formation of the PARP1/Timeless complex, blocked the recruitment of Timeless to DNA break sites, and limited HR repair to a low level even in RIOX1-depleted cells (Fig. 5f-h). These results suggest that SGF29 is involved in RIOX1-modulated HR repair by interacting with K491me-bearing cGAS to prevent cGAS from binding to PAR.

### Human RIOX1 modulates HR repair by regulating the methylation of the corresponding human cGAS K506

Human cGAS contains a conserved K506 site corresponding to K491 in mouse cGAS (Fig. S3B). To further investigate whether the RIOX1-mediated mechanism is species- or cell type-specific, we validated our aforementioned findings in human oral keratinocytes (HOKs). As expected, knockdown of RIOX1 by shRNAs enhanced HR repair after exposure to ionizing radiation (Fig. S5A). Human cGAS was capable of interacting with RIOX1 and SET7 (Fig. S5B and S5C). By using a validated antibody recognizing human cGAS K506me (Fig. S5D), we found that loss of RIOX1 largely increased the level of cGAS K506me and induced the association between cGAS and SGF29 in HOKs (Fig. S5E and S5F). In contrast, the basal cGAS K506me was totally abolished by knockdown of SET7 (Fig. S5G). Furthermore, the cGAS K506R or SGF29 D194A/D196 A mutation promoted the binding of cGAS with PARP1 and abrogated the interaction between PARP1 and Timeless (Fig. S5H–S5J). These results suggest that the RIOX1-guided regulation of HR repair is conserved between human and mouse cells.

### RIOX1-modulated cGAS K491me regulates the radiosensitivity of bone marrow cells

**Fig. 6:**
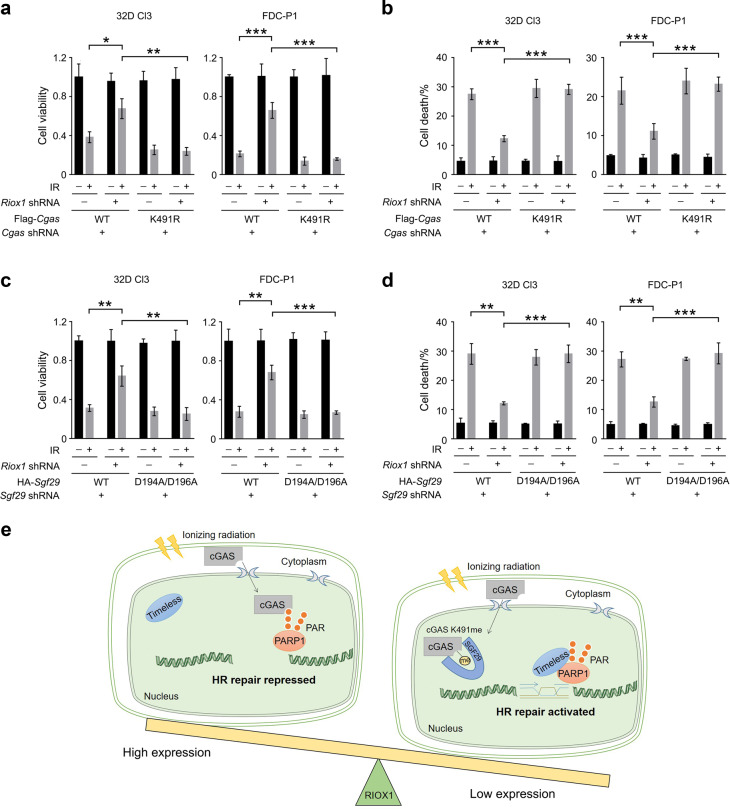
RIOX1-mediated cGAS K491me and the cGAS/SGF29 interaction regulate the radiosensitivity of bone marrow cells. **a−d** The data are shown as the mean ± SD of three independent assays. *P < 0.05; **P < 0.01; ***P < 0.001. **a**, **b** 32D Cl3 and FDC-P1 cells expressing *Riox1* shRNA, *Cgas* shRNA, WT Flag-*Cgas*, or Flag-*Cgas* K491R were treated with 10 Gy ionizing radiation. Cell viability **a** and cell death **b** were evaluated 48 h after irradiation. Cell viability data were normalized to the untreated group. **c**, **d** 32D Cl3 or FDC-P1 cells expressing *Riox1* shRNA, *Sgf29* shRNA, WT HA-*Sgf29*, or HA-*Sgf29* D194A/D196A were treated with 10 Gy ionizing radiation. Cell viability **c** and cell death **d** were evaluated 48 h after irradiation. Cell viability data were normalized to the untreated group. **e** Schematic of RIOX1-mediated repression of HR repair

To determine the biological impact of cGAS K491me and the cGAS/SGF29 interaction on bone marrow cells, we knocked down endogenous cGAS or SGF29 in RIOX1-intact or RIOX1-depleted 32D Cl3 and FDC-P1 cells and exogenously expressed the cGAS K491R or SGF29 D194A/D196A mutant. Although loss of RIOX1 substantially increased cell viability and reduced cell death after irradiation, these effects were largely abolished by the cGAS K491R or SGF29 D194A/D196A mutation (Fig. 6a-d). Together, these results suggest that RIOX1-modulated cGAS K491me regulates the radiosensitivity of bone marrow cells.

## Discussion

Ionizing radiation-induced DNA damage is cytotoxic and becomes lethal when it is not repaired properly and effectively.^25^ RIOX1 was previously identified in a proteomic screen as a binding partner of the transcription factor Osterix.^9^ Ablation of RIOX1 promoted bone formation in mice, leading to increased bone mass and density.^26^ In the present study, we demonstrated that both bone marrow cells isolated from RIOX1 knockout mice and *Riox1* shRNA-treated bone marrow-derived cell lines showed increased viability after exposure to ionizing radiation. Accordingly, the efficiency of HR repair, rather than NHEJ repair, was markedly enhanced in RIOX1-depleted cells. Therefore, our data highlight a novel role of RIOX1 in regulating the radiosensitivity of bone marrow cells.

Lysine methylation and demethylation are dynamic cyclical processes, and a well-balanced protein lysine methylation status is a prerequisite for diverse biological processes, including DNA repair.^22^ In this study, by using in vitro methylation/demethylation assays, we demonstrated that cGAS K491 can be monomethylated by SET7 and that this methylation is removed by RIOX1. Although SET7 can catalyze the methylation of multiple nonhistone proteins,^27^ to the best of our knowledge, cGAS is the first nonhistone demethylation substrate discovered for RIOX1. More importantly, we demonstrated that RIOX1 is highly expressed in bone marrow tissues and bone marrow-derived cell lines, accompanied by a low cGAS K491me level. In contrast, the expression level of SET7 fluctuates only minimally among different tissues and organs. Therefore, the present data suggest that the expression of RIOX1 is the dominant factor controlling the cGAS K491me level.

cGAS is a cytosolic DNA sensor.^28^ Either exogenous or endogenous DNA can trigger cGAS-dependent production of cGAMP, which in turn activates STING to elicit innate immune responses.^19^ Recently, cGAS was found to translocate into the nucleus in response to ionizing radiation. Independent of its nucleotidyltransferase activity, nuclear cGAS binds to the PAR chain of PARP1 and competitively inhibits PARP1-mediated recruitment of Timeless to DNA damage sites, retarding the process of HR repair^18^. In this study, we further deciphered how the interaction between cGAS and PAR is regulated by protein methylation. We found that the 483–497 aa region at the C-terminus of the cGAS protein, which contains the K491 methylation site, is responsible for cGAS binding with PAR. The methyllysine reader protein SGF29 binds to K491me-bearing cGAS and masks, at least partially, the PAR binding motif, thereby sequestering cGAS and preventing it from interacting with PAR (Fig. 6e). Therefore, our current data add to the current knowledge of the nonimmune function of cGAS in DNA repair.

As previously reported, JMJD-containing demethylases, such as JMJD1A, JHDM2A and JMJD5, modulate DNA repair efficiency through genome-wide histone modifications.^6–8^ The present study demonstrates a novel RIOX1-mediated mechanism in which RIOX1 acts as a nonhistone demethylase to govern cGAS K491me and consequently modulates the HR repair machinery at DNA break sites. Furthermore, the correlation of high RIOX1 expression and low cGAS K491me and the increased survival rate of RIOX1-depleted bone marrow cells underscore the critical role of RIOX1 in the regulation of cellular radiosensitivity. The present data suggest that RIOX1 inhibitors might be potential bone marrow-protective agents during radiotherapy.

## Materials and methods

### Materials

The rabbit polyclonal anti-cGAS K491me antibody was produced by Boer Biotechnology (Chengdu, China). The mouse cGAS K491me peptide was synthesized and injected into rabbits. An affinity column with bound nonmodified peptide was used to collect and purify rabbit serum, excluding antibodies targeting nonmethylated cGAS. The antibody was then purified using an affinity column linked with bound cGAS K491me peptide. A rabbit polyclonal antibody recognizing human cGAS K506me was produced following the same protocol.

Antibodies recognizing cGAS, Tubulin, GST, His, SET1A, SET1B, SET7, MLL1, MLL3, γH2A.X and SMYD3 were obtained from Cell Signaling Technology. Antibodies recognizing RIOX2 were purchased from Atlas Antibodies. Antibodies recognizing RIOX1, ASH1L, PARP1, MLL2, SGF29, Timeless, NSD1, NSD2, H2A.X, CD11b, CD11a, CD34, Thy-1, and SMYD2 were purchased from Abcam. The antibody against Flag, anti-Flag M2 agarose beads, streptavidin-conjugated agarose beads, biotin, bovine serum albumin and poly(ADP-ribose) glycohydrolase (PARG) recombinant protein were purchased from Sigma. Horseradish peroxidase-conjugated goat anti-mouse and anti-rabbit secondary antibodies were purchased from Thermo Fisher Scientific. Biotin (terminal)-PAR Polymer was purchased from Trevigen.

### DNA constructs and mutagenesis

The PCR-amplified mouse *Cgas*, *Riox1*, *Riox2*, *Set7*, *Timeless* and *Sgf29* and human cGAS, SET7, Timeless and SGF29 sequences were subcloned into the indicated vectors. A QuikChange Site-Directed Mutagenesis Kit (Stratagene, La Jolla, CA) was used to generate the mutant constructs.

The following shRNAs were used in this study: scrambled shRNA, GCTTCTAACACCGGAGGT CTT; mouse *Riox1* shRNA-1, TTTTTAATAAATCTGACGT (recognizing a noncoding sequence); mouse *Riox1* shRNA-2, TATCATATAACATGGTTGC; mouse *Cgas* shRNA, TTTCAGAAG GCAATGTCAG (recognizing a noncoding sequence); mouse *Set7* shRNA-1, AACAGTATTAGGTCCAACT; mouse *Set7* shRNA-2, AATCCGTCATCGTCCAGGT; mouse *Sgf29* shRNA, TATTTGATCATAGGAACTC (recognizing a noncoding sequence); mouse *H3f3b* shRNA, ACAAATGCAGTCTAGTCAG (recognizing a noncoding sequence); human cGAS shRNA, AGTTCTTACTGAAAAACAG (recognizing a noncoding sequence); and human SGF29 shRNA, ACGCAGGTCTGTGATCATC (recognizing a noncoding sequence). Other shRNAs used in this study were obtained from Sigma.

### Cell culture and stable cell line establishment

32D Cl3 and FDC-P1 cells were maintained in RPMI 1640 medium containing 10% fetal bovine serum (FBS). C2C12 and NIH/3T3 cells were cultured in Dulbecco’s modified Eagle’s medium (DMEM) containing 10% FBS. AML12 cells were grown in DMEM:F12 medium supplemented with 10% FBS. MLE 12 cells were maintained in HITES medium supplemented with 2% FBS. HOKs were obtained from ScienCell (Carlsbad, CA) and were maintained in Oral Keratinocyte Medium (Carlsbad, CA) supplemented with 10% fetal bovine serum.

To generate stable cell lines with depletion of gene expression, cells were transfected with lentiviral vectors carrying shRNAs and subsequently selected with puromycin. To generate stable cell lines with gene overexpression, cells with silencing of endogenous gene expression were transduced with lentiviral vectors carrying the indicated gene sequence and were then selected with hygromycin.

### Mice irradiation and bone marrow cell isolation

Irradiation of six-week-old C57BL/6 mice was performed with a ^137^Cs gamma-ray source. A single dose of radiation was administered at 1 Gy per min. Mice were then sacrificed, and tibiae were harvested. An EasySep™ Mouse Hematopoietic Progenitor Cell Isolation Kit (STEMCELL Technologies) was used to isolate hematopoietic progenitor cells; Dynabeads™ Mouse Pan B (Thermo Fisher Scientific) was utilized to isolate B cells; and an EasySep™ Mouse Neutrophil Enrichment Kit (STEMCELL Technologies) was utilized to isolate neutrophils. Animals were treated according to the relevant institutional and national guidelines and regulations. The use of animals was approved by the institutional review board of West China Hospital of Stomatology.

### Cell viability assay

A Cell Counting Kit 8 (WST-8/CCK-8) was purchased from Abcam, and the assay was performed following the manufacturer’s protocol.

### Cell death assay

Cell death was assessed by trypan blue staining as reported previously.^29^

### Generation of RIOX1 knockout mice

RIOX1 knockout mice were generated via CRISPR/Cas9 gene editing by GemPharmatech.

### Protein expression in bacteria and protein purification

BL21(DE3) cells expressing cGAS, cGAS, cGAS, RIOX1, SET7, SGF29 and the corresponding mutant proteins were maintained in LB medium and induced with isopropyl β-D-1-thiogalactopyranoside (IPTG) for 16 h at 30 °C before lysis via sonication. Purification of the tagged proteins was performed as reported previously.^30^

### Analyses of DNA repair efficiency

NHEJ and HR repair were analyzed based on previously reported methods^13^, and a schematic of this assay is shown in Fig. S1G. In brief, 32D Cl3 or FDC-P1 cells were transfected with the DR-GFP plasmid, which was unable to express DR-GFP because of the I-SceI site. Expression of I-SceI induced a DSB in the DR-GFP locus. The PCR products were amplified with primers targeting the sequences flanking the I-SceI site and were then double digested with the enzymes I-SceI and BcgI. NHEJ repair caused loss of the I-SceI site and made the PCR product resistant to double digestion with I-SceI and BcgI, producing a 0.65-kb PCR band. HR repair caused the I-SceI-digested sequence to be replaced with a BcgI cut site, restoring the expression of GFP.

DR-GFP-expressing cells were immediately cotransfected with a vector expressing I-SceI. After transfection for 36 h, to inhibit DNA repair and promote the accumulation of I-SceI-mediated DSBs, the cells were treated with NU7441 (1 μmol·L^**−**1^) and KU55933 (10 μmol·L^**−**1^) for 12 h and were then exposed to ionizing radiation. To measure the NHEJ repair efficiency, cells were synchronized in G1 phase by double thymidine block, and genomic DNA was extracted 1 h after ionizing radiation to quantify the 0.65-kb PCR product. To measure the HR repair efficiency, cells were stained with Hoechst 72 h after ionizing radiation. The numbers of GFP-expressing and Hoechst-positive cells were counted under a fluorescence microscope to calculate the ratio of GFP-positive cells to total cells. At least 10 fields, each containing more than 100 cells, were counted. To measure the efficiency of I-SceI cleavage, genomic DNA was extracted immediately after irradiation. The amount of 0.65-kb PCR product generated from the naive, I-SceI-uncut DNA was used as an indirect reference value for I-SceI-induced DSBs. β-Actin was used as the internal control. The primer sequences were as follows: DR-GFP-F, 5-CTGCTAACCATGTTCATGCC-3; DR-GFP-R, 5-AAGTCGTGCTGCTTCATGTG-3; β-actin-F, 5- GACAGGATGCAGAAGGAGATT ACTG; β-actin-R, CTCAGGAGGAGCAATGATCTTGAT.

### ChIP-PCR assay

As reported previously, an Upstate Biotechnology kit was used to perform the ChIP-PCR assay.^30^ The specific primers used for PCR were designed to target the sequence 0.2 kb away from the I-SceI-induced DSBs: 5-GATCAGGCAGAGCAGGAACC-3 (forward) and 5-GAACAGCTCCTCGCC CTTGC-3 (reverse).

### Comet assay

Total bone marrow cells were isolated from Riox1 knockout mice or WT mice and subjected to 10 Gy ionizing radiation. The comet assay was performed using a Single Cell Gel Electrophoresis Assay/Comet Assay (R&D Systems) following the manufacturer’s protocol.

### In vitro methylation assay

Purified SET7 protein (500 ng) and purified cGAS protein (200 ng) were incubated in 100 μL of methylation buffer at 30 °C for 1 h according to a previously reported method.^31^ SDS loading buffer was added to stop the reaction, and immunoblot analysis was then conducted.

### In vitro demethylation assay

Purified RIOX1 protein (500 ng) and purified cGAS protein (200 ng) were incubated with demethylase reaction buffer at 37 °C for 1 h, as mentioned previously.^32^ SDS loading buffer was added to stop the reaction, and immunoblot analysis was then conducted.

### cGAS activity assay

cGAS activity was detected by using a Transcreener cGAMP cGAS TR-FRET Assay Kit (BellBrook Labs) following the manufacturer’s instructions.

### Diarrhea severity assessment

Animals were subjected to irradiation for 5 days, and diarrhea severity was recorded according to a previously defined grading system^33^.

### Statistical analysis

Unless specifically indicated, significant differences were analyzed by two-tailed unpaired Student’s *t* test. Unless otherwise specified, all data are presented as the mean ± SD of three independent experiments/samples.

## Supplementary information

RIOX1 Suppl infos

Fig S1

Fig S2

Fig S3

Fig S4

Fig S5
