# Supplementary material for: RIOX1-demethylated cGAS regulates ionizing radiation-elicited DNA repair
Source: Bone Res. 2022 Feb 24;10:19. doi: 10.1038/s41413-022-00194-0 (PMC8873214; doi:10.1038/s41413-022-00194-0)
Supplement: Supplementary file 1 — RIOX1 Suppl infos [file 41413_2022_194_MOESM1_ESM.docx]

**Supplementary figure legends**

**Figure S1 RIOX1 dampens cell viability and HR repair in bone marrow cells after ionizing radiation**

(A) The body weight and limb length of WT and RIOX1 knockout mice (n = 7) were measured at birth. *, *P* < 0.05; **, *P* < 0.01.

(B) The body weight and body length of WT and RIOX1 knockout mice (n = 7) were measured at 2 months of age. *, *P* < 0.05; **, *P* < 0.01.

(C) WT and RIOX1 knockout mice (n = 7) were exposed to 9 Gy ionizing radiation, and the survival curves were plotted. P value was calculated using the log-rank test.

(D) The symptom of diarrhea was measured in WT and RIOX1 knockout mice (n = 7) exposed to 9 Gy ionizing radiation. **, *P* < 0.01.

(E) Immunoblot was performed using the indicated antibodies.

(F) The whole bone marrow cells isolated from WT or *Riox1* knockout mice were exposed to 10 Gy ionizing radiation. Comet assay was performed 30 min after irradiation. Scale bar, 15 µm.

(G) Schematic of measurement of HR and NHEJ repair.

(H-I) 32D Cl3 and FDC-P1 cells were transfected with I-SceI-expressing vector, and cells were exposed to 10 Gy ionizing radiation 72 h after transfection. HR pathway was measured by counting the ratio of GFP-positive cells under fluorescent microscope (H). I-SceI cleavage efficiency was measured immediately after irradiation (I). Scale bar, 80 µm.

**Figure S2 A non-histone substrate is involved in RIOX1-repressed HR repair**

(A-C) Immunoblotting was performed with the indicated antibodies.

(A) 32D Cl3 cells with expression of *Riox1* shRNA, WT Flag-*Riox1*, or Flag-*Riox1* H302A/H367A were transfected with I-SceI-expressing vector. HR pathway was measured 72 h after transfection. *Riox1* shRNA targets the non-coding region. Exo, exogenous; Endo, endogenous.

(B) 32D Cl3 cells with expression of *Riox1* shRNA, WT Flag-*Riox1*, or Flag-*Riox1* H302A/H367A were exposed to 10 Gy ionizing radiation.

(C) 32D Cl3 cells with expression of *H3f3b* shRNA, Flag-*H3f3b* K4R (upper panel), or Flag-*H3f3b* K36R (bottom panel) were transfected with I-SceI-expressing vector. HR pathway was measured 72 h after transfection. *H3f3b* shRNA targets the non-coding region.

**Figure S3 RIOX1 demethylases cGAS K491me**

(A) Flag-Riox1 was expressed in 32D Cl3 cells. Flag-Riox1 immunoprecipitates were subjected to mass spectrometry analyses. Selected peptide hits of cGAS-associated proteins identified by mass spectrometry analyses are shown.

(B) Alignment analysis of the spanning sequence of cGAS K491, H3K4 and H3K36 among species.

(C) Dot-blot analyses of the anti-cGAS K491me antibody against the His-tagged unmodified and modified cGAS K491 peptides.

(D) 32D Cl3 cells were expressed with shRNA targeting the indicated genes. Immunoblotting was performed with the indicated antibodies.

(E) 32D Cl3 cells were expressed with *Riox1* shRNA. Immunoblotting was performed with an anti-cGAS K491me antibody in the presence of blocking peptide.

(F) 32D Cl3 cells with expression of *Riox1* shRNA were exposed to 10 Gy ionizing radiation. Immunoblotting was performed.

**Figure S4 RIOX1 minimally affects the activity or subcellular distribution of cGAS**

(A-C) Immunoblotting was performed with the indicated antibodies.

(A) 32D Cl3 cells with expression of *Riox1* shRNAs were exposed to 10 Gy ionizing radiation.

(B) Purified Flag-cGAS protein was incubated with purified His-SET7 protein for an *in vitro* methylation assay. cGAS activity was measured in presence or absence of HT-DNA.

(C) 32D Cl3 cells with expression of *Riox1* shRNAs were exposed to 10 Gy ionizing radiation. Nuclear or cytoplasmic fraction was prepared. C, cytosol; N, nucleus.

(D) A schematic of the measurement of proteins recruited to I-Scel cleavage site.

**Figure S5** **Human RIOX1 modulates HR repair by regulating the corresponding human cGAS K506me**

(B-J) Immunoblotting was performed with the indicated antibodies.

(A) HOK cells with expression of hRIOX1 shRNAs were transfected with I-SceI-expressing vector. HR pathway was measured 72 h after transfection.

(B-C) Immunoprecipitation with indicated antibodies were performed using the lysates derived from HOK cells.

(D) Dot blots were performed in presence of indicated peptides.

(E, G) HOK cells were expressed with indicated shRNAs

(F) HOK cells were expressed with hRIOX1 shRNA, and immunoprecipitation with indicated antibodies were performed.

(H) HOK cells with expression of hRIOX1 shRNA, WT Flag-hcGAS or Flag-hcGAS K506R were exposed to 10 Gy ionizing radiation. Immunoprecipitation was performed. The immunoprecipitates were treated with excessive purified PARG protein before subjected to immunoblot analyses.

(I) HOK cells with expression of hRIOX1 shRNA, hcGAS shRNA, WT Flag-hcGAS, Flag-hcGAS K506R, or His-hTimeless were exposed to 10 Gy ionizing radiation. A Ni-NTA pulldown and immunoprecipitation was performed. The precipitates were treated with excessive purified PARG protein before subjected to immunoblot analyses. cGAS shRNA targets the non-coding region.

(J) HOK cells with expression of hRIOX1 shRNA, Flag-hcGAS, His-hTimeless, hSGF29 shRNA, WT HA-hSGF29, or HA-hSGF29 D194A/D196A were exposed to 10 Gy ionizing radiation. A Ni-NTA pulldown and immunoprecipitation was performed. The precipitates were treated with excessive purified PARG protein before subjected to immunoblot analyses. SGF29 shRNA targets the non-coding region.
