# Supplementary figures and images for: RIOX1-demethylated cGAS regulates ionizing radiation-elicited DNA repair
Source: Bone Res. 2022 Feb 24;10:19. doi: 10.1038/s41413-022-00194-0 (PMC8873214; doi:10.1038/s41413-022-00194-0)

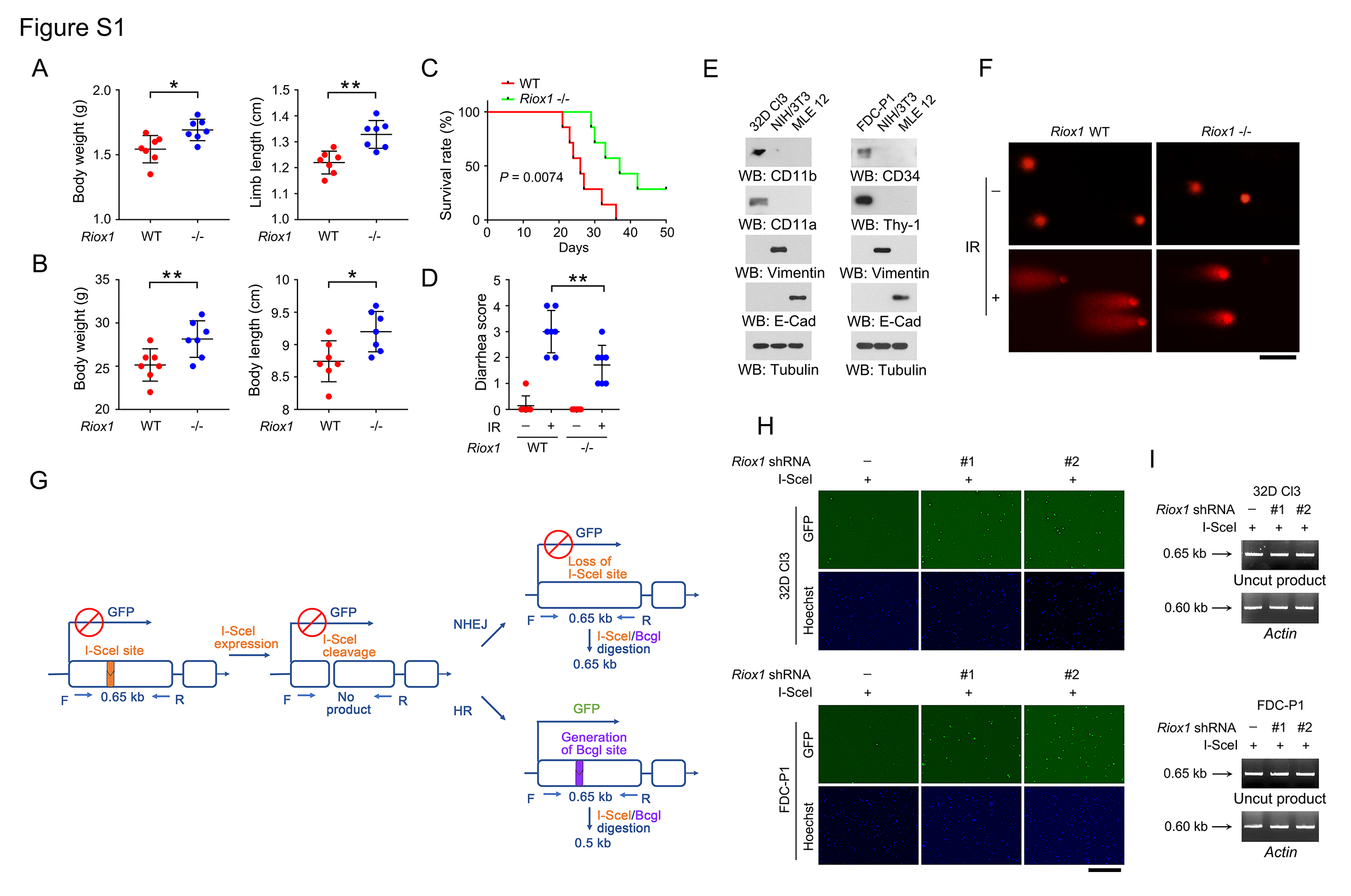

Supplement: Supplementary file 2 — Fig S1 [file 41413_2022_194_MOESM2_ESM.tif]

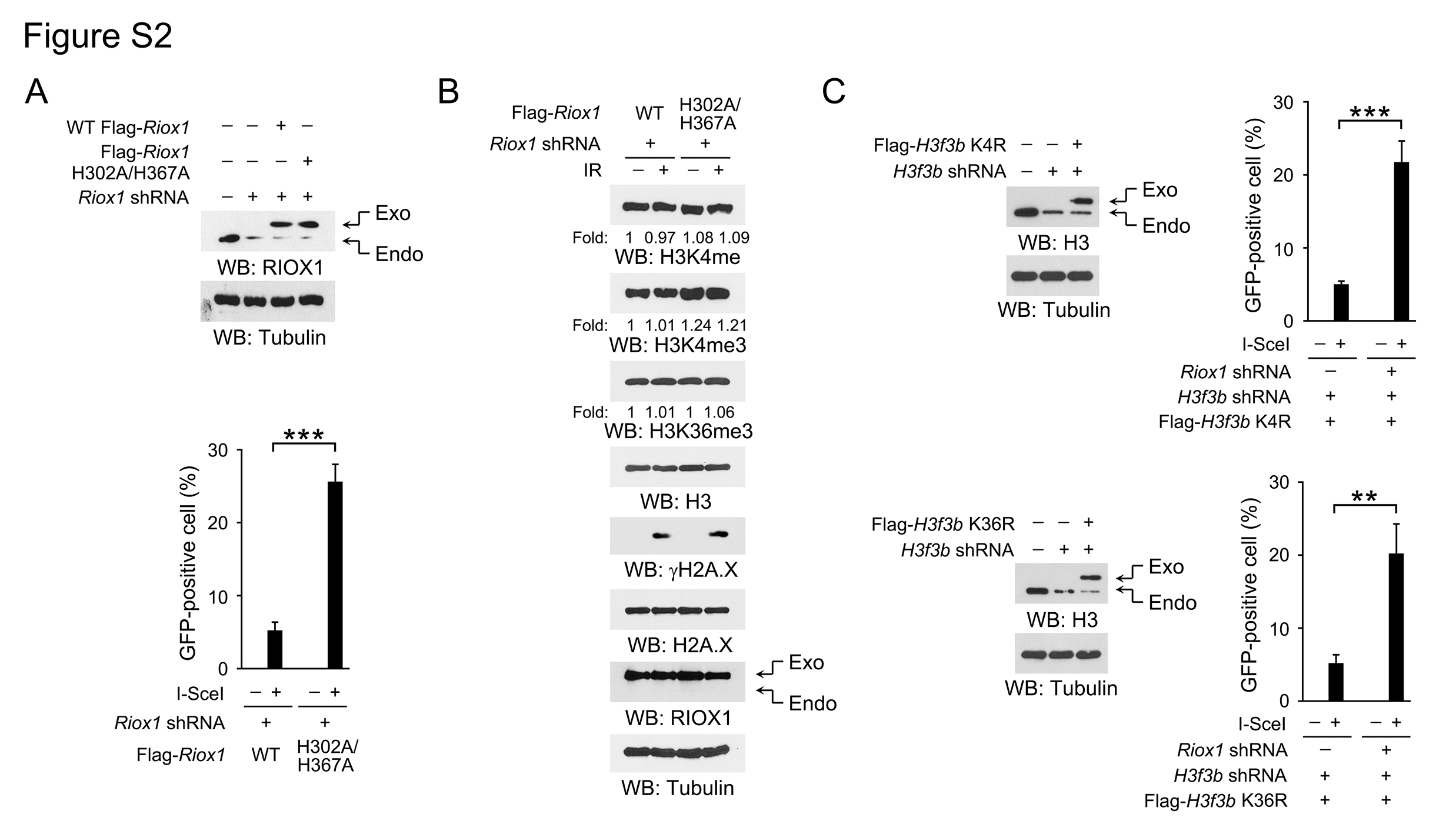

Supplement: Supplementary file 3 — Fig S2 [file 41413_2022_194_MOESM3_ESM.tif]

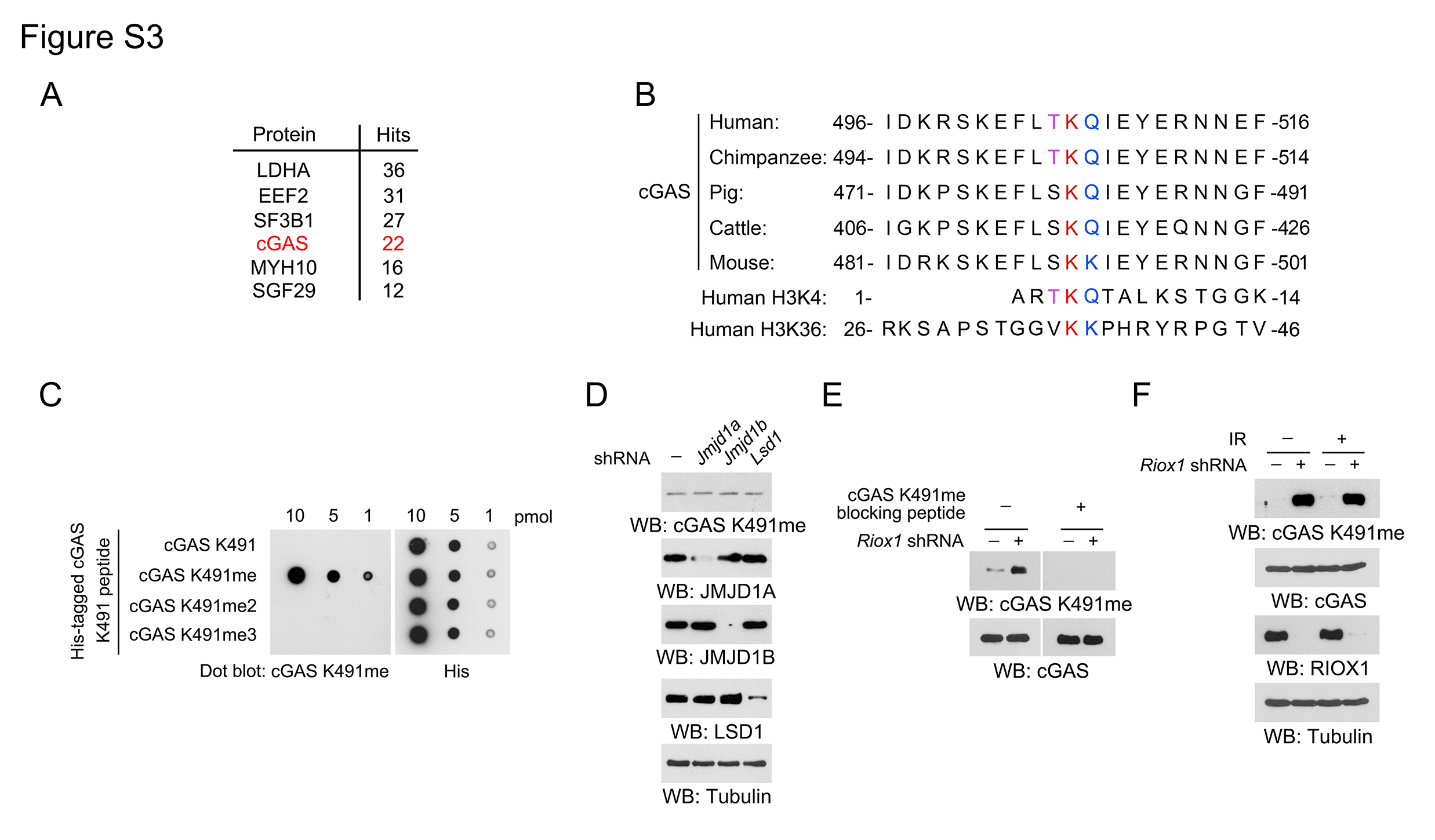

Supplement: Supplementary file 4 — Fig S3 [file 41413_2022_194_MOESM4_ESM.tif]

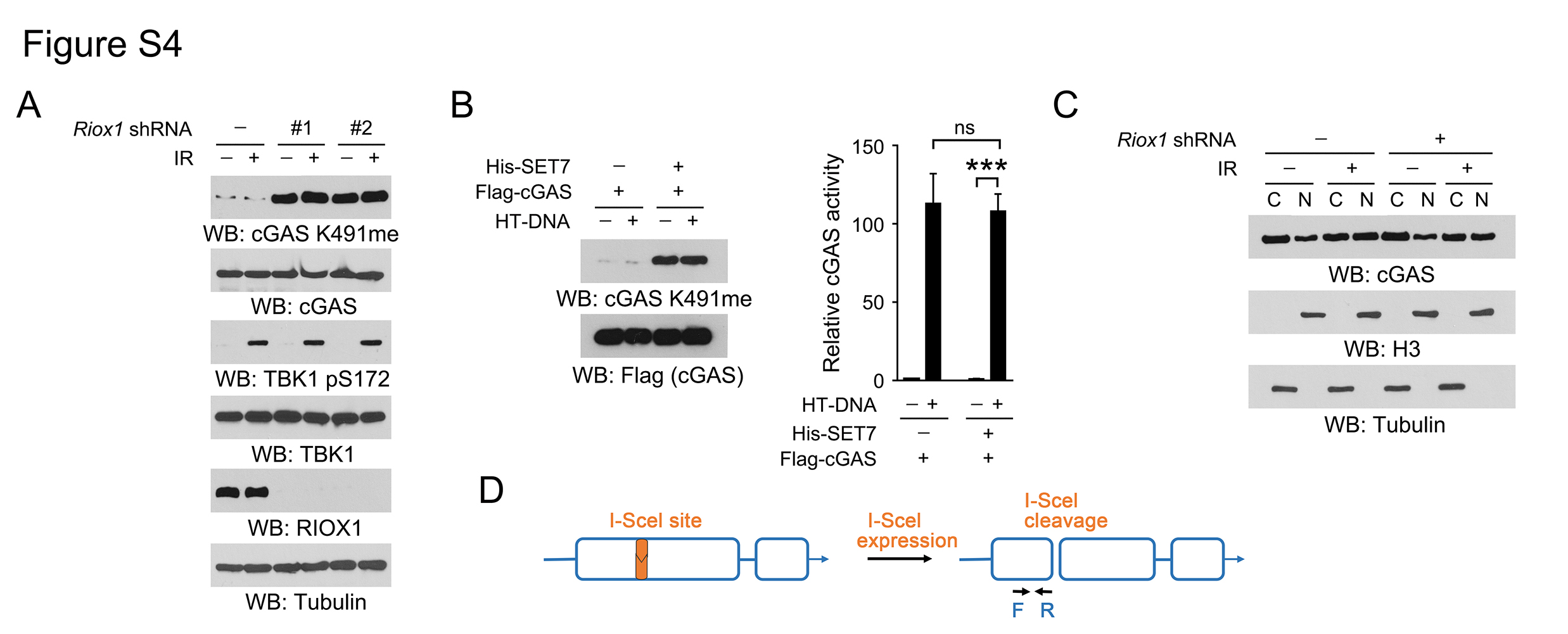

Supplement: Supplementary file 5 — Fig S4 [file 41413_2022_194_MOESM5_ESM.tif]

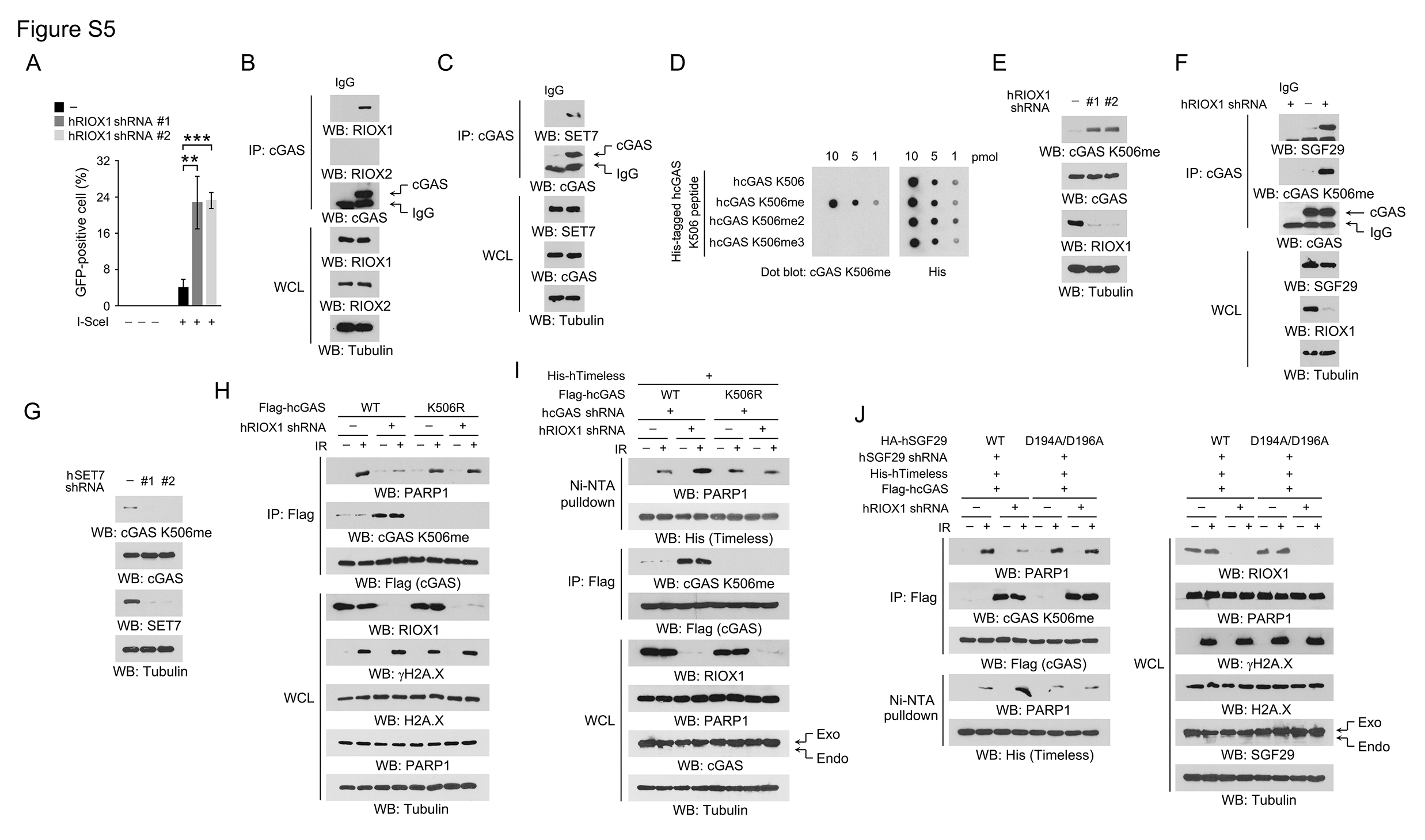

Supplement: Supplementary file 6 — Fig S5 [file 41413_2022_194_MOESM6_ESM.tif]
